# Hidden mycota of pine needles: Molecular signatures from PCR-DGGE and Ribosomal DNA phylogenetic characterization of novel phylotypes

**DOI:** 10.1038/s41598-018-36573-z

**Published:** 2018-12-21

**Authors:** Rajesh Jeewon, Quin S. Y. Yeung, Dhanushka N. Wannasinghe, Sillma Rampadarath, Daneshwar Puchooa, Hong-Kai Wang, Kevin D. Hyde

**Affiliations:** 1 Department of Health Sciences, Faculty of Science, University of Mauritius, Reduit, Mauritius; 2 School of Biological Sciences, Faculty of Science, University of Hong Kong, Pokfulam Rd, Hong Kong, SAR China; 3 Center of Excellence in Fungal Research, Mae Fah Luang University, Chiang Rai, 57100 Thailand; 4 Faculty of Agriculture, University of Mauritius, Réduit, Mauritius; 5 Biotechnology Institute, Zhejiang University, 310029 Zhejiang, P. R. China; 6 Key Laboratory for Plant Biodiversity and Biogeography of East Asia (KLPB), Kunming Institute of Botany, Chinese Academy of Science, Kunming, 650201 Yunnan, China

**Keywords:** Genetics, Fungi

## Abstract

Previous studies for enumerating fungal communities on pine needles relied entirely on phenotypic diversity (microscopy) or identification based on DNA sequence data from those taxa recovered via cultural studies. To bypass limitations of the culturing methods and provide a more realistic diversity estimate, we employed and assessed a PCR-DGGE based method coupled with rDNA phylogenetic sequence analyses to characterize fungal taxa associated with pine needles. Fresh (living) and decayed needles from three hosts of the Pinaceae (*Keteleeria fortunei*, *Pinus elliottii* and *P. massoniana*) were examined. Morphological studies reveal that the most abundant species associated with decayed needles were *Cladosporium cladosporioides* and an unidentified *Trichoderma* species followed by *Gliocephalotrichum* sp., *Gliocladium* sp., *Lophodermium pinastri, Paecilomyces varioti*, *Phaeostalagmus cyclosporus* and a *Phoma* sp, which are commonly occurring fungi. Community genomic data from freshly collected and decayed pine needles recovered 40 operational taxonomic units, which appear to be mostly undetected members of the natural fungal consortium. Sequence analyses revealed a number of phylotypes or “species” that were not recovered using traditional morphological and cultural approaches previously used. Phylogenetic data from partial 18S rDNA sequence data reveal that most phylotypes represent potential novel phylogenetic fungal lineages with affinities to the Dothideomycetes, Leotiomycetes, Lecanoromycetes and Sordariomycetes and were not identical to previously known endophytes or saprobes. Although the major ecological roles of these phylotypes in pine needles are still enigmatic, this study provides new insights in hidden fungal diversity that mycologists are possibly ignoring given the discrepancies associated with available methods. To what extent do previously recovered identified species (either as saprobes or endophytes) from morphological or culturing studies act as pioneer decomposers or constitute an integral part of endophytic community warrants further investigation.

## Introduction

Fungi, especially from pines, are important contributors to primary productivity and nutrient cycling. They are mostly isolated as endophytes, pathogens or saprobes and are critical components in plant ecosystems^1–3^. Several studies have focused on the characterization of fungi colonising pine needles from ecological and pathological perspectives (e.g.^4–10^). The pioneering work on mycota of pine litter by Kendrick and Burges^4^ prompted other researchers to focus on this host, which is an important component of many forests. Traditional methods documenting fungal diversity, however, rely mostly on taxonomic identification of species based on spore morphology and DNA sequence data of endophytes from cultural dependent methods. This has a major drawback given that not all fungi grow readily in highly selective medium and sporulation is dependent on species, host, seasonality, growth conditions, and other environmental factors which can largely underestimate diversity.

In many cases, fungi exist predominantly as vegetative hyphae, which make microscopic examination difficult^3,7^. In addition, the use of artificial media does not reflect natural conditions and only those microorganisms that thrive well under these conditions will grow. It is thought that only a small fraction (0.1 to 10%) of microorganisms existing in the nature can be cultured artificially^11^. The use of previous classical methods to enumerate fungal taxa or characterise fungal communities from pine needles has therefore underestimated diversity. Generally, most studies were limited to temperate regions and have consistently isolated only a few dominant fungi, the most common of which are *Lophodermium pinastri* and *L. piceae*^12^. Recently, several studies based on DNA sequence analyses, especially those targeting endophytes, have also revealed different groups of fungal species not recovered via traditional methods (e.g.^8–10^).

To circumvent the challenges associated with spore production and cultivation, a number of molecular based approaches have been widely used (as detailed in Anderson *et al*.^13^). Among these, PCR-DGGE has proven to be a powerful technique to detect and characterize fungal communities in soil samples, plant material (roots and leaves), rocks, water, alga and glass^13–17^. Studies have revealed that dominant microorganisms isolated from environmental samples using traditional and cultivation-dependent methodology are genetically different from those identified via DGGE analysis of rDNA gene (e.g.^18^). Diversity of freshwater fungi in decaying leaves of white birch (*Betula papayrifera*), several maple species (*Acer rubrum*, *A. saccharum*, and *A. spicatum*) and white spruce (*Picea glauca*) has been reported using a traditional and a molecular approach^19^. They found that species richness estimates based on DGGE are higher than those from T-RFLP analysis and much higher than those based on spore identification. Subsequent DGGE analyses of aquatic hyphomycetes have also revealed that presence or absence of fungal taxa and biomass from community DNA is regulated primarily by season and type of substrates^20^. Based on sequence analyses of DGGE bands, Vainio & Hantula^15^ successfully detected a higher fungal diversity from environmental samples of *Picea abies* (Norway spruce stumps) than when using culture techniques. Cullings *et al*.^21^ used DGGE to evaluate changes in soil communities in response to the effects of artificial defoliation of pines in a pine-spruce forest. From a pathological perspective, DGGE has also been valuable in profiling fungal taxa that are associated with healthy and infected black spruce seedlings (*Picea marina*)^22^.

Our previous studies on pine needles from *Keteleeria fortunei*, *Pinus elliottii* and *Pinus massoniana* (Family: Pinaceae) in Hong Kong revealed a few taxa new to science^23,24^ and a large number of unidentified taxa, especially those that exist predominantly in their asexual stages and sterile hyphae. To the best of our knowledge, there is no known studies on pine needles of these 3 members of the Pinaceae that have analysed the fungal species (especially those colonizing internal tissues) based on PCR-DGGE followed by 18S rDNA sequence analyses. Mahajan *et al*.^9^ have attempted to characterize microbial communities from *Pinus roxburghii* of the Himalayan subtropical region (India) based on PCR-DGGE using ITS-GC clamped primers but all PCR amplifications failed and no fungal taxa could be sequenced. Aneja *et al*.^25^ used DGGE to characterize bacteria and fungi but their focus was detection of potential saprobic fungi that are potential decomposers in spruce planted *in vitro*. In this study we attempt to recover fungal species found in pine needles using a polyphasic approach (direct morphological comparison, cultural based methods and PCR-DGGE analyses of 18S rDNA coupled with phylogenetic analyses). The objectives of this study were to (1) profile the main fungal species associated with different decay stages and (2) find out whether fungal species from pine needles are more diverse than expected and, (3) whether “taxa” recovered from the molecular approach are related to previously identified endophytes or whether they constitute unknown phylogenetic lineages.

## Results

### Morphological-based methodology

A total of 39 taxa were identified from the three hosts and 2 needle types (Table 1). A large number of anamorphic (asexual) fungi (77%) were recovered, while the average occurrence of sexual morph ascomycetes was quite low (6.25%). The most abundant taxa were *Cladosporium cladosporioides*, *Gliocephalotrichum* sp., *Gliocladium* sp., *Paecilomyces varioti*, *Phaeostalagmus cyclosporus*, *Phoma* sp., and a *Trichoderma* sp. The percentage of non-sporulating taxa was 14.58%. A proportion of fungi (14.5%) existed as cultures that could not be identified as no sporulating structures were observed. These were termed as “Mycelia sterilia” (*sensu*^3,26^) and have the highest frequency of occurrence. *Lophodermium pinastri* has the highest frequency of occurrence among the three ascomycetes present. Anamorphic fungi were dominant on both, L and F needle types (with 100% dominance on *Keteleeria fortunei* (L-type needles), 94.74% on *K*. *fortunei* (F-type needles), 96.77% on *Pinus elliottii* and 93.75% on *P*. *massoniana*).

**Table 1:** Average frequency of occurrence of fungi in the L-type needles of *Keteleeria fortunei*, *Pinus elliottii* and *Pinus massoniana* and F-type needles of *Keteleeria fortunei*.

Average frequency of occurrence in various hosts	*Keteleeria fortunei* (L-type needles)	*Keteleeria fortunei* (F-type needles)	*Pinus elliottii* (L-type needles)	*Pinus massoniana* (L-type needles)
*Acremonium* sp.	0.71	0	0	0
*Beltraniella ondinae*	0	0	0.71	0
*Bionectria* sp.	0	0.71	0	0
*Bostrichonema* sp.	0	0	45.71	65.71
*Cancellidium pinicola*.	0	0	0	0.71
*Chaetopsina* sp.	0.71	0	0	0
*Cladosporium cladosporioides*	53.57	22.14	87.86	45
*Unidentified Coelomycete*	2.14	2.86	7.86	10.71
*Coleophoma oleae*	0	0.71	0	0
*Colletotrichum* sp.	0	0	3.57	5.71
*Crytophiale guadalcanalensis*	0	0	4.29	19.29
*Curvularia inequalis*	0	0	2.14	0
*Curvularia lunata*	0	0	0	3.57
*Embellisia allii*	0	0	0.71	0
*Gliocephalotrichum* sp.	42.14	32.86	13.57	42.86
*Gliocladium* sp.	31.43	35.71	18.57	55.71
*Lophodermium pinastri*	0	0	36.43	9.29
*Microdochium* sp.	0	0	7.86	2.14
*Mycoenterolobium* sp.	0	0	0	1.43
*Myrothecium* sp.	2.14	1.43	2.14	5
*Nigrospora oryzae*	0	0	3.57	0
*Unidentified Oomycetes*	0.71	0	5.71	1.43
*Paecilomyces varioti*	25.71	5.71	12.86	17.86
*Penicillium* sp.	0.71	0	0	0.71
*Pestalotiopsis bicilia*	0	0	0	6.43
*Pestalotiopsis conigena*	0	0	1.43	0
*Pestalotiopsis disseminata*	0	0	8.57	0
*Pestalotiopsis espaillatii*	0	0	10.71	9.29
*Pestalotiopsis montellica*	0	0	5	0
*Pestalotiopsis olivacea*	0.71	0	10	10
*Phaeostalagmus cyclosporus*	20	19.29	7.14	5
*Phialocephala dimorphospora*	0.71	0	0	0
*Phoma* sp.	2.14	0.71	5.71	3.57
*Unidentified* mould	2.86	2.14	15.71	20
*Sordaria lappae*	0	0	0	0.71
*Thozetella nivea*	0	0	6.43	2.14
*Thozetella pinicola*	18.57	6.43	16.43	1.43
*Thozetella tocklaiensis*	0	0	6.43	0.71
*Thysanophora penicilioides*	0.71	0	0	0
*Trichoderma* sp.	69.29	67.86	63.57	82.86
Mycelia sterlia sp. 1	0.71	0	0	4.29
Mycelia sterlia sp. 2	10	15	0	4.29
Mycelia sterlia sp. 3	0	0	0	4.29
Mycelia sterlia sp. 4	4.29	20	0	10.71
Mycelia sterlia sp. 5	12.86	13.57	4.29	0
Mycelia sterlia sp. 6	71.43	54.29	74.29	78.57
Mycelia sterlia sp. 7	38.57	21.43	11.43	15.71
Zygomycete (lower fungi) sp.	0.71	0.71	0	0
Species Diversity Index	1.08	1.04	1.21	1.20

The highest species diversity as calculated by Shannon diversity index was recorded from L-type needles on *Pinus elliottii* (1.21), followed by *P*. *massoniana* (L-type) (1.20), *Keteleeria fortunei* (L-type) (1.08) and the lowest was on *K*. *fortunei* (F-type) (1.04). Cluster analyses (Bray-Curtis similarity) and multi-dimensional scaling (MDS) were applied to show the similarities and differences of the fungal communities on *Keteleeria fortunei*, *Pinus elliottii* and *P. massoniana*. The L-type needles and F-type needles of *Keteleeria fortunei* were similar in fungal diversity. Samples were interspersed in three different clades rather than forming distinct clades based on needle types. Dendograms generated also indicated that species composition from *Pinus elliottii* and *P*. *massoniana* were different from those of *Keteleeria fortunei*. Samples from *Pinus elliottii* and *P*. *massoniana* (both F and L needle types) are assembled in one cluster whereas samples from *Keteleeria fortunei* (both F and L needle types) were found to form two different clusters without any relationships to needle types. Results also reveal that species composition from L-type needles of *Pinus elliottii* and *P*. *massoniana* was very similar and have similar fungal diversity but clades corresponding to either host were not observed. In addition, we also noted that there was no clear relationship between fungal diversity and seasonal variation among *Pinus elliottii* and *P*. *massoniana* (results not shown). For instance, samples collected from winter (Nov-Mar) and summer (May-Sep) seasons were found in all the clades. However, in the case of *Keteleeria fortunei*, samples collected in winter appeared in distinct clades and were distant from those collected in summer months.

### Molecular-Based Methodology

#### DGGE analyses

**Figure 1:**
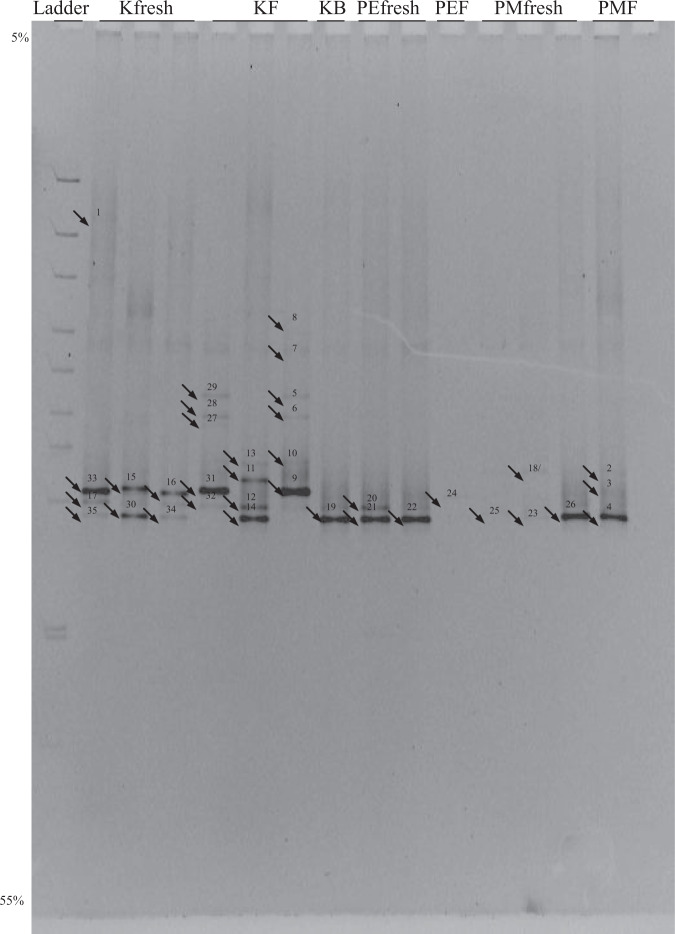
The first DGGE analyses of the fungal sequences amplified from pooled DNA samples from different decay stages of needles from Pinaceae species. Lane 1 is the ladder; Lanes 2–8 are samples from *Keterleeria fortunei* with lanes 2–4 from fresh needles, lanes 5–7 from L-type needles and lane 8 from F-type needles. Lanes 9–11 are samples from *Pinus elliotti* with lanes 9–10 from fresh needles and lane 11 from L-type needles. Lanes 12–16 are the samples of *Pinus massoniana* with lanes 12–14 from fresh needles and lanes 15–16 from L-type needles.

**Figure 2:**
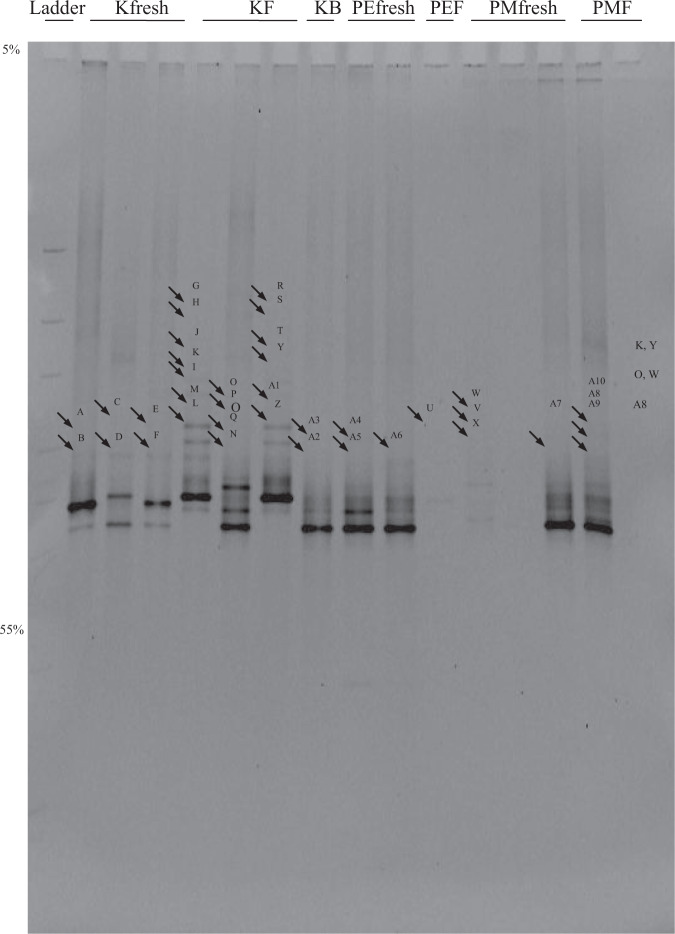
The second DGGE analyses of the fungal sequences amplified from pooled DNA samples from different decay stages of needles from Pinaceae species. Lane 1 is the ladder; Lanes 2–8 are samples from *Keterleeria fortunei* with lanes 2–4 from fresh needles, lanes 5–7 from L-type needles and lane 8 from F-type needles. Lanes 9–11 are samples from *Pinus elliotti* with lanes 9–10 from fresh needles and lane 11 from L-type needles. Lanes 12–16 are the samples of *Pinus massoniana* with lanes 12–14 from fresh needles and lanes 15–16 from L-type needles.

At least two sets of DGGE profiles were generated in order to ensure the results obtained were reproducible. All bands that migrated at different positions were sequenced to ensure that the maximum number of phylotypes was identified. This also enabled us to compare whether bands migrating at different positions are phylogenetically similar or dissimilar. The most appropriate denaturant gradient, which gave better resolution, was 5–55% urea/formamide. The first DGGE analysis revealed 35 bands (Fig. 1) while the second one revealed 36 bands (Fig. 2). Most of the bands found in Fig. 1 are also present in Fig. 2 but there were a few exceptions: bands 1, 2, 3, 10, 17 and 35, present in the first gel (Fig. 1) were not observed in the second gel (Fig. 2). In addition, bands G, H, V, W, Z, A3, A8 and A9 were present in the second gel but not in the first gel. Bands were selected and excised based on their positions and mobility from both gels. Forty-four bands were sequenced and 39 were successful (87% success).

Some bands were weak but this did not affect the quality of sequences as these bands were purified and reamplified again prior to sequencing. Fungal diversity of *Keteleeria fortunei* (especially on L-type needles) was the highest among the three Pinaceae species as more taxa were detected. Eight, 16 and 2 bands were recovered from fresh (living), L-type needles and F1-type needles of *Keteleeria fortunei* respectively. DGGE of DNA samples from *Pinus massoniana* resulted in 9 bands (4 and 5 from living and L-type needles respectively). The lowest phylotypic diversity was recorded from *Pinus elliottii* (3 and 1 from living and L-type needles respectively).

#### Sequence analysis

Based on blast search results, 30 phylotypes (75%) were similar to known bitunicate fungi (Dothideomycetes); 2 were similar to the Eurotiales; 5 similar to the Letiomycetes and 1 to the Lecanoromycetes; 1 was a unitunicate taxon and similar to the Hypocreales. All sequences display reasonable similarity (94% or more) to fungal sequences in GenBank. Those having highest sequence similarity were included in the phylogenetic analyses. Analyses based on different criteria yielded essentially similar trees and the phylogram generated from Bayesian analyses with bootstrap support generated under Maximum Likelihood is presented here. Some bands present in one needle type were also found in the other needle types. For example, bands B, D and F from L-type needles of *Keteleeria fortunei* share similar mobility gradient as bands A5 and A6, recovered from fresh needles of *Pinus elliottii*. However, blast search results and phylogenies indicate that co-migrating bands are not necessarily similar or related. For instance, bands A or 33 (Figs 1 and 2 respectively; from the living needles of *Keteleeria fortunei*), M or 31 (from L-type needles of *K. fortunei*) and 3 (Fig. 1; from L-type needles of *Pinus massoniana*) were found to be at the same position but sequence analyses revealed that they were totally different taxa. Band M or 31 was found to be highly similar and related to the Rhytismatales (Letiomycetes), while band A or 33 was similar and related to *Cochliobolus sativus* (Pleosporales) and band 3 was similar and related to an uncultured clone, whose classification within the Dothideomycetes, is unknown. Similar results were obtained with bands 17, L (or 32) and A4 (or 20), which migrated to the same position on the gel. Co-migrating bands were also found within one needle type but results also indicate that they are not be similar or related. For instance, bands O (or 13) and 10 were from L-type needles of *Keteleeria fortunei* but sequence comparison revealed that band O (or 13) and 10 were dissimilar and phylogenetically unrelated. Band O is related to an uncultured clone (Dothideomycetes) whereas band 10 was a sister taxon to the Rhytismatales. Similar results were obtained with bands A (or 33) and C (or 15) recovered from living needles of *Keteleeria fortunei* and L (or 32) and Q (or 12) from L-type needles of *Keteleeria fortunei*. This phenomenon clearly indicates that bands (phylotypes) co-migrating at the same position have distinct evolutionary linages. This is not surprising as it has already been demonstrated in previous studies that phylogenetically distant taxa can have co-migrating bands and that one band does not necessarily mean one unique phylotype^27–29^.

**Figure 3:**
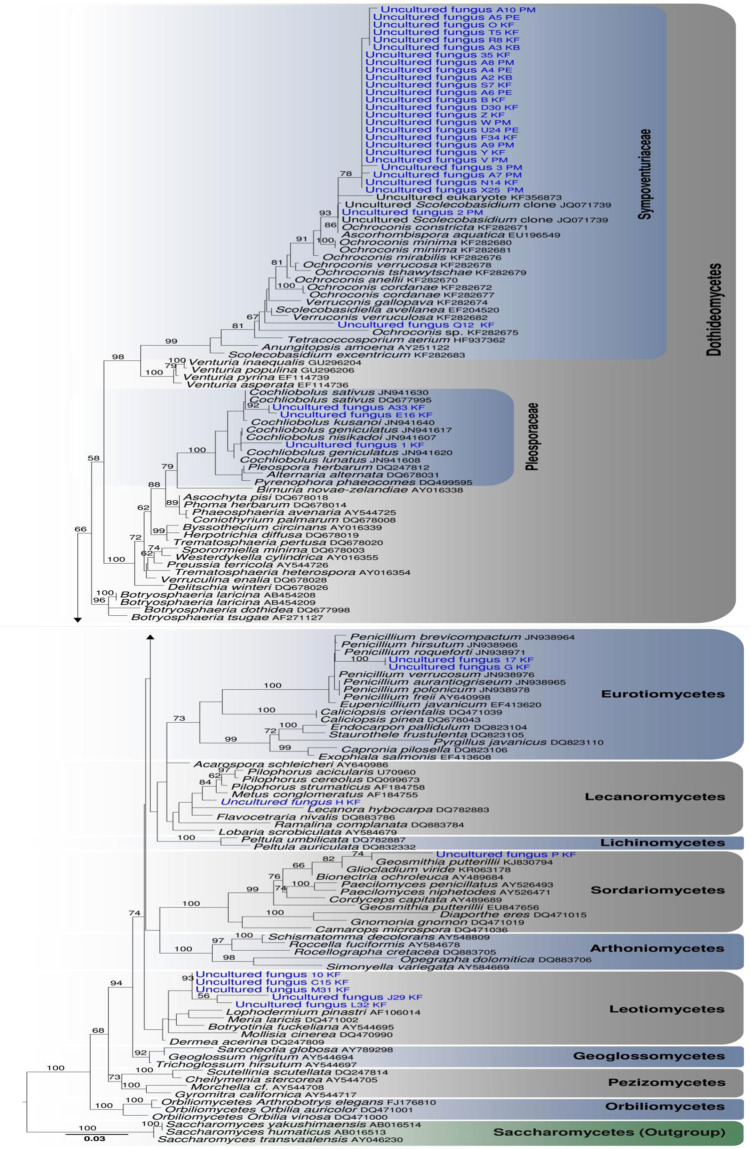
Phylogram generated from Bayesian analysis of partial 18S rDNA sequence data. *Saccharomyces* taxa are outgroups. Bootstrap support generated under Maximum Likelihood criterion are shown on the branches. Uncultured isolates are in Bold and First alphabet corresponds to the DGGE band whereas alphabets in brackets correspond to the host from which the isolates were obtained from. Ex. Uncultured fungus V (KF) means “DGGE band V from *Keetalaria fortunei*”. PM = *Pinus massoniana*; PE = *P. elliottii*.

Sequences obtained from respective DGGE bands were analysed under different optimality criteria as stated before. All trees generated yielded same topologies with regards to the position of our phylotypes (except in one ML analysis where the position of the Eurotiomycetes and Dothideomycetes were slightly different but this relationship was unstable with no support). The Bayesian tree, rooted with *Saccharomyces* is selected to infer phylogenetic position of our phylotypes (Fig. 3). Phylogeny shows that most of the phylotypes sequenced form a distinct and moderately supported monophyletic group (78% BS) basal to other known bitunicate ascomycetes, especially to the family Sympoventuriaceae (Fig. 3). In all phylogenies, phylotype 2 PM clusters with a few cultured *Scolecobasidium* clones with strong support while phylotype Q12 KF is nested in between *Verruconis* and *Ochroconis* species. Phylotypes A (33), E (16), and 1 KF belong to the family Pleosporaceae and are closely related to *Cochlobolius sativus* and related species (Pleosporaceae). Phylogram also depicts that our two phylotypes (G KF and 17 KF) constitute an independent and strongly supported subclade (100% BS) within the genus *Penicillium* (Eurotiales, Eurotiomycetes) while uncultured isolate H KF is related to *Metus conglomeratus* and *Lecanora hybocarpa* and is best accommodated within the Lecanorales. The unitunicate phylotype P KF clusters with *Geosmithia putterilli* with moderate support and also shares close affinities to *Gliocladium viride* (Hypocreales). Phylotypes 10 KF, C15 KF, M31 KF, J29 KF and L32 KF have close phylogenetic affinities to other anamorphic Leotiomycetes (especially *Lophodermium pinastri* and other relatives of the Rhytismataceae, Rhytismatales).

## Discussion

### Morphology based diversity studies

Anamorphic fungi were dominant and accounted for approximately 80% of the total fungal taxa. This is largely consistent with previous morphological studies where anamorphic fungi constituted the bulk of fungal communities within pine needles^25^. This abundance of anamorphic taxa indicates that the cultural techniques tend to favour faster growing anamorphic fungi, which outcompete others. Fungal taxa with the highest frequency of occurrence were those that failed to produce any spores. They were categorised as mycelia sterila and not sequenced herein as these endophytes are rather well documented (e.g.^5,6,30^) and the main aim in this study was to use PCR-DGGE to investigate if pine needles harbour further potential unknown fungal taxa. Common saprobes, such as *Cladosporium cladosporioides* and *Trichoderma* sp., were also high in occurrence. These two taxa have previously been reported to be dominant and pioneer decomposers of *Pinus*^4,30–33^.

In this study, *Pinus elliottii* was found to have the highest fungal species richness among the three-tree species. This figure is much lower than other plant substrates such as grasses and palms but results corroborate other diversity studies on pine needles (e.g.^7^). Eighty fungal taxa were identified from two *Pandanus* species (*Pandanus furcatus* and *P*. *tectorius*) of the family *Pandanaceae*^34^ and 205 fungal species were identified from *Gramineae* and *Cyperaceae*^35^. Tokumasu^36^ reported that the number of fungal species recorded from the L-type needles of *Pinus densiflora*, *P*. *sylvestris*, *P*. *banksiana*, *P*. *rigida* and *P*. *strobus* were 26, 12, 20, 26 and 22 respectively. Pine needle morphology and structure may account for this lower diversity. The presence of thick waxy cuticles covering the needles is a major characteristic of the Pinaceae. Penetration of cuticle is the crucial step for fungal invasion of the leaves and this waxy cuticle is a primary defense against microbial invasion^37^.

*Keteleeria fortunei*, *Pinus elliottii* and *P*. *massoniana* yielded 26, 31 and 30 taxa, respectively. Twenty taxa were common on all tree species. For *Keteleeria fortunei*, species richness in F-type needle was lower than L-type needles (19 as compared to 25) and this supports other studies that fungal diversity is usually higher in L-type needles^36^. One explanation to account for this difference is that fungi in L-type needles are mostly those occurring at leaf senescence (possibly endophytes). Another explanation might be related to the amount of sugars present within pine needles. Millar^38^ suggested that there are inadequate sugar residues within F-type needles as compared to L-type and therefore less nutrient availability. This phenomenon has already been demonstrated in numerous other studies (e.g.^33,36,39^). A similar species richness between *Pinus elliotti* and *P. massoniana* can be explained by the fact that both hosts are related, have similar needle morphology and are from the same location. Fungal composition of needle litter within *Pinus* species have also been reported to be similar^32,33,36^. *Lophodermium pinastri* (Leotiomycetes) was only found in *Pinus elliottii* and *P*. *massoniana* but not in *Keteleeria fortunei*. It is possible that this fungal species, as stated in^12^, is restricted to *Pinus* but no other tree species in the family Pinaceae. Our phylogenetic data based on uncultured sequences, however, seem to contradict what Minter^12^ postulated. Based on sequence analyses in this study, it cannot be confirmed that we recovered *L. pinastri* but at least four phylotypes from *K. fortunei* from fresh and L type needles that were related to the Leotiomycetes. Our molecular data clearly demonstrate that diversity of Letiomycetous fungi is higher than expected. Phylogenies also indicated that phylotypes from the same host or needle types (either fresh or decayed) were not necessarily related.

### Molecular Based Diversity Studies

#### Phylotype diversity and community shift

DGGE fingerprints suggest that fungal diversity was high in the living (fresh) and during early needle decay (L type) whereas only 1–2 bands could be detected in the F type needles. This suggests that only 1–2 fungal phylotypes are dominant during that particular decay stage (F type). A higher phylotypic diversity was also observed in *Keteleeria fortunei* (Figs 1 and 2). In most cases, we found that more bands (with clear signals and more conspicuous) were recovered from the L-type needles, except in *Pinus ellioti*. DGGE banding profiles also revealed a diversity shift of fungal 18S rDNA from fresh to L and F decay stages. This obviously indicates that the fungal community was initially represented by more fungi in the living and L type needles and then diversity of the fungal community gradually decreases as the needles start to decay. As further decomposition of pine needles occurs (F-type needles), only 1–2 abundant fungi dominate and this indicates a community shift from one that is highly diverse to one with a reduced number of abundant fungi (e.g.^25^). The latter are presumably those that have greater role in the decomposition process but such an assumption needs to be verified. It should be noted, however, that such a diversity shift and distinct phylotypes associated with different decay stages have not been found in the other two *Pinus* species investigated. Based on available information, it is very difficult to account for this difference. It might be that (i) phenolic compounds found in large amounts in *Pinus* needles could have been inhibitory to DNA and PCR amplication^40^ or (ii) limitations in our molecular approach (e.g. DGGE resolution) that failed to recover other taxa^41,42^.

#### Phylogeny of phylotypes and comparison with phenotypic diversity

To improve our understanding of the relationship between diversity and taxonomy, we generated phylogenies and made an attempt to identify and classify the uncultured phylotypes. Phylotypes 17 and G isolated from fresh and L type needles respectively cluster together with high support, and are fungi related and belong to *Penicillium*. The latter are common asexual fungi that have also been obtained from cultural studies in high frequency (Table 1) and similar findings have also been reported^9,43^. Phylotype P belongs to the order Hypocreales and could possibly be a *Geosmithia* species given their close phylogenetic affinity. Jankowiak *et al*.^44^ reported nine unnamed species of *Geosmithia* that are associated with bark beetle species infecting pine branches while Kolařík *et al*.^45^ reported *Geosmithia* as either saprobes or pathogens. Our phylotype herein is presumably a saprobe as it was collected from F type needles but the possibly that it is an endophyte and behaves as a pathogen cannot be ruled out as endophytes are known to adopt different ecological strategies (e.g.^2^). Taxa from the Eurotiales and Hypocreales have been recovered herein given their high abundance, ubiquitous nature and are also commonly recovered from microbial communities associated with healthy and diseased spruce seedlings^22^.

A common endophytic genus from pine needles is *Lophodermium*, a leotiomycetous fungus of the order Rhytismatales^6,32,36,46^. Based on previous studies, we have hypothesised that *Lophodermium* would be the predominant taxon but such was not the case. Surprisingly only 5 phylotypes (10 KF, C15 KF, M31 KF, J29 KF and L32 KF) constitute an independent lineage and share a close relatedness to *Lophodermium pinastri, Meria laricis, Botryotinia fuckelina* and *Mollisia cinerea*, which are members of the Rhytismatales and Helotiales (Leotiomycetes, Fig. 3). Even whether these phylotypes are *Lophodermium* species is doubtful and our results corroborates those of Ono *et al*.^47^ who reported few OTU’s isolates of the Leotiomycetes from pines. Numerous endophytic studies on pine needles based on cultural methods have recovered *Lophodermium* species (e.g^5^). It has generally been assumed that *Lophodermium* species, given their abundance, play an important and primary role in needle decomposition. However, are *Lophodermium* species pioneer decomposers? It could be possible that the large number of phylotypes in this study could also be a significant contributor to decomposition. However, function of most of them is still elusive and it is impossible to draw real conclusions as to their role. It is highly possible that they perish soon after needle senescence, as some of them were not recovered from cultural and morphological studies. For those similar taxa that were recovered from fresh and decayed needles, the assumption that they change their lifestyle (i.e. change from endophytic mode to saprophytic mode) to enable them to make maximum use of available nutrients or to flourish in other ecological niches cannot be overlooked. Such a competitive strategy has already been demonstrated in leaves of *Magnolia lillifera*^2^.

Contrary to our expectation, DNA sequence data analysed demonstrate that the majority of phylotypes from living leaves and L type needles (A10 PM-X25 PM) constitute two major clades close to other bitunicate taxa (Dothideomycetes) especially to *Ochroconis*, *Scolecobasidium*, and *Ascorhombispora*, which belongs to the family Sympoventuriaceae (Venturiales). Could these phylotypes represent isolated lineages that can provide clues to unaccounted fungal diversity and potential ecological roles? Phylogeny indicates that this novel monophyletic lineage has accumulated more nucleotide substitution than other bitunicate taxa and it might be possible that these uncultured taxa diverged recently. It is, for the time being, difficult to propose any taxonomic rank (Class, Order, or Family) to accommodate this new lineage, given that the systematics of its sister taxa are unclear and unresolved.

Another interesting finding is the detection of a particular phylotype (H KF) that has a close phylogenetic affiliation to *Lecanora hybocarpa, Flavocetraria nivalis, Metus conglomeratus* and *Pilophorus* species (order Lecanorales). To date, there are no reports of any lichenised fungi closely related to those genera from pine needles. This clearly shows that DGGE analyses allow (i) recovery of other leotiomycetous and lichenised fungi that possibly previously escaped morphological detection and (ii) suggest that diversity among these groups of fungi within pine needles is higher than expected. Many taxa of the Pleosporales appear to be quite common from environmental samples^6,21,22^. DGGE analyses on community DNA from rhizosphere of diseased seedling of *Picea mariana* (Black spruce) recovered three environmental clones that were related to *Paraphaeosphaeria* and *Leptosphaeria* (Pleosporales)^22^. Another uncultured clone related to *Phoma herbarum* (Pleosporales) was isolated from cankers of Spruce seedling. Pleosporales fungi are economically important plant pathogens and there is a high probability therefore that these phylotypes are predominantly endophytes and colonise pine needles without causing any apparent disease. Our molecular data corroborate with those obtained from^6^ who recovered eight environmental clones from needles of *Pinus taeda* that were related to *Alternaria* (Pleosporales). Our molecular approach recovered three phylotypes that fits within the Pleosporaceae and related to *Cochlobolius* species which are closely related to *Pleospora herbarum*. It is worth pointing out that our morphological studies also yielded identified taxa such as *Curvularia* species (anamorphs of *Cochliobolus*), *Embellisia* and *Phoma* (anamorphic *Alternaria*) but it is difficult to ascertain whether the DGGE phylotypes in this study were any of these taxa (but it cannot be ruled out). Another major discrepancy noted was the absence of *Cladosporium* from our molecular approach but this was abundant from our morphological survey as well as from environmental clones^6^. In addition, no basidiomycetes and xylariaceous taxa were recovered here but these were common from *Pinus taeda*^6^.

#### Significance and limitations of the study

Our molecular approach therefore, indicates that the majority of taxa are not necessarily related to those isolated from conventional techniques. Arnold *et al*.^6^ also reported that a number of operational taxonomic units recovered were distinct from those obtained from the cultural studies. We presume that those “DNA based taxa” might play a more active ecological and functional role in the community, whereas those that are easily identified from the cultural and morphological approach represent only selective endophytes- those that colonise aerial parts or inner surfaces and those that are fast growing on selective media but not necessarily abundant in the tissues. Apart from inadequate knowledge about the precise ecological nature and function of these phylotypes, this study raises a few questions about the reliability of DGGE and only one molecular approach in documenting the diversity of natural fungal consortia. Although primers targeting partial ribosomal DNA (this and other studies) appear to detect fungal DNA from environmental samples, they usually amplify only a short fragment that is either too conserved (e.g. 18S rDNA) or too variable (e.g ITS rDNA). Only one primer pair which recovers about 350 bp amplicons was tested and it appears to be taxonomically biased and too specific as no basidiomycetes or other fungal groups (apart from Ascomycetes) were recovered. This study could have therefore reflected only a fraction of those cryptic taxa within pine needles. The small number of bands recovered could possibly be linked to (i) the limited resolution of DGGE (ii) individual bands that were masked and possibly contain more than one phylotypes, and (iii) specificity of primers and size of DNA fragment amplified. In bacterial diversity studies, it has been shown that different DNA protocols and purification methods yield different DGGE profiles^48^. Similar research are required in fungal studies in addition to the use of a combination of different primer pairs that would target a wider group of fungi. Another intriguing finding was the abundance of taxa that constitutes new phylogenetic lineages. Given that only DNA sequences or other genomic data are available for uncultured “taxa”, an appropriate nomenclatural system and “species” definition based on DNA sequence characters are also critical issues that need to be dealt with. Potential bias of rDNA primers and application of DGGE in estimating fungal diversity have already been detailed^49^ and are not discussed here.

#### Future avenues with regards to environmental sequences

Phylogenetic information obtained here cannot be linked to function but it does provide insights into the relative abundance of uncultured phylotypes or “taxa” that could be more functional and metabolically active than those obtained from morphological and cultural methods. It is obvious that fungal diversity knowledge is still rudimentary. Our study reveals that even small substrates, such as pine needles harbour a diversity of enigmatic and cryptic operational taxonomic units (potential novel species) that can be recovered only by a molecular approach. We suspect that there could be a myriad of more cryptic taxa. Given that the roles of these phylotypes are still enigmatic, we should not only rely on findings based on taxonomic characterization but the way forward would be to proceed with metagenomic sequencing to enable analyses of functional gene content and properly understand community structure. Future work should also target to look into possible avenues about how to induce those taxa to sporulate in culture, describe their morphology, assess their physiological characteristics and exploit their potential ecological and functional roles. While our molecular approach herein permits recovery of potential new phylotypes, we are also fully cognizant of the intricacies associated with reliability of the 18S rDNA region and possibly more gene regions with greater sequence variability (e.g. ITS) and their drawbacks should be investigated to provide a more realistic picture of mycobiota. Should we consider those “loner” sequences/environmental phylotypes with distinct lineages as neglected properties or orphans simply because we cannot give them scientific names? There is a need to translate those molecular sequences into formal taxonomic names and consider them nomenclaturally valid to facilitate taxonomy, as postulated by Hibbett^50^ and Jeewon *et al*.^51^ but with caution^52^.

## Experimental Procedures

### Morphological based methods

#### Collection sites and substrates

Two study sites in Hong Kong (Cape D’ Aguilar and Sai Kung Yung Shue O) and senescent needles were collected randomly from the ground from three hosts (*Keteleeria fortunei*, and *Pinus elliottii* and *P. massoniana*) at 2 monthly intervals over a year. Decaying needles were categorised as L-types (senescent needles recently fallen light brown and occurring in an uncompacted layer at the surface) and F-types (grey to black needles, with softened tissues, low tensile strength and a higher moisture content than L-type needles forming a more closely packed stratum) as described by Kendrick and Burges^4^.

#### Plating technique

To document fungi, a modified washing and plating technique of Tokumasu^36,53^ was used for the treatment of needles. Five needles were placed into centrifuge tubes with the addition of sterilized 0.005% Aerozol OT solution (Di-iso-octyl sodium sulfosuccinate). The capped tubes were shaken vigorously in a vortical shaker at 300 oscillations per minute for one minute. Solutions were then discarded and the procedure repeated five times. Needles were then washed with sterile water three times and dried on sterilised filter paper for one day (to prevent bacterial growth). Needles were then placed onto the surface of half-strength cornmeal agar plates (only one needle in one agar plate) and incubated at room temperature (~24 °C). The samples were examined weekly over 1–2 months to check for any developing sporulating structures under the microscope. Fungal species diversity one each tree species was calculated using the Shannon Diversity index (H^1^).

### Molecular Based Methods

#### DNA extraction and amplification

Living needles (green needles still attached to branches), L-type needles and F-type needles of *Keteleeria fortunei* and Living needles and L-type needles of *Pinus elliottii* and *Pinus massoniana* were also collected from Cape D’ Aguilar. All the samples (at least 3 samples from each needle type) were kept in paper bags and stored in the fridge at 4 °C and worked on the following days. Every single needle was cut into segments and ground with liquid nitrogen (with 1% PVPP) and DNA extracted following Jeewon *et al*.^54^ but using hot phenol which yielded better DNA suitable for amplification. The 5′end of the 18S ribosomal DNA (rDNA) was amplified with the primer pairs NS1 (5′-GTA GTC ATA TGC TTG TCT C-3′) and fungal-specific oligonucleotide GCfung (5′-CGC CCG CCG CGC CCC GCG CCC GGC CCG CCG CCC CCG CCC CAT TCC CCG TTA CCC GTT G-3′) as suggested by May *et al*.^55^. One PCR reaction mixture included 5 μl of 10x PCR buffer, 4 μl of 10 μM dNTPs, 0.5 μl of 0.1% bovine serum albumin (BSA), 0.5 μl of 1% PVP, 0.3 μl Taq polymerase, 31.7 μl nanopure water, 1.5 μl of 10 μM of each primer, 3 μl of 30 ng of each DNA sample. PCR profile was as follows: Initial denaturation for 3 min at 95 °C followed by 35 amplification cycles at 95 °C for 1 min, 50 °C for 1 min and 72 °C for 3 min, followed by a final extension of 72 °C for 10 min. PCR products were used for DGGE analysis.

#### DGGE analysis

An aliquot of 25 μl of each PCR product was mixed with and equal amount of dye and loaded in urea/formamide (100% denaturing solution with 7M urea and 40% formamide) denaturing gradient in a 7% acrylamide gel and run at 150 V in 1x TAE buffer (pH 8) at 60 °C (DGGE-2001, CBS Scientific Co.). The denaturing gradient was first optimised using a range of denaturant concentrations and electrophoresis with time. Gradient of 5–55% urea/formamide was selected based on a series of optimisation experiments. The gel was stained in 1x TAE containing ethidium bromide (EtBr) for 30 min and de-stained in 1x TAE for 20 min. The gel images were captured under UV trans-illumination and analysed by the computer program Gel Doc. Bands were excised and each fragment was placed into a 1.5 ml marked microcentrifuge tubes. They were reamplified with the original primers and purified using a DNA purification kit (Amersham Biosciences GFXTM PCR DNA and Gel Band Purification Kit) prior to automated sequencing using NS1 primer (API Prism 3730, Applied Biosystems). DGGE analyses were performed twice under the same conditions.

#### Phylogenetic analyses

Partial 18S rDNA gene sequences recovered from DGGE were aligned in Bioedit^56^, in MAFFT v. 7 with the web server (http://mafft.cbrc.jp/alignment/server) with similar sequences from different fungal orders^57–60^. Alignments were also checked and edited manually to optimise alignment. Phylogenetic analyses were conducted in PAUP version 4.0b10 as described by^61–63^. In addition to Maximum Parsimony, alternative analyses based on the maximum likelihood (ML) and Bayesian analyses (BI) were also conducted (as described in^64–66^). Phylograms were visualised with FigTree v. 1.4.0 and annotated in Microsoft PowerPoint (2007) as described in^65^.

## Supplementary information

Supplementary information.
