# Author Correction: Hidden mycota of pine needles: Molecular signatures from PCR-DGGE and Ribosomal DNA phylogenetic characterization of novel phylotypes

**DOI:** 10.1038/s41598-020-68364-w

**Published:** 2020-07-15

**Authors:** Rajesh Jeewon, Quin S. Y. Yeung, Dhanushka N. Wannasinghe, Sillma Rampadarath, Daneshwar Puchooa, Hong-Kai Wang, Kevin D. Hyde

**Affiliations:** 1 Department of Health Sciences, Faculty of Science, University of Mauritius, Reduit, Mauritius; 2 School of Biological Sciences, Faculty of Science, University of Hong Kong, Pokfulam Rd, Hong Kong, SAR China; 3 Center of Excellence in Fungal Research, Mae Fah Luang University, Chiang Rai, 57100 Thailand; 4 Faculty of Agriculture, University of Mauritius, Réduit, Mauritius; 5 Biotechnology Institute, Zhejiang University, Zhejiang, 310029 People’s Republic of China; 6 Key Laboratory for Plant Biodiversity and Biogeography of East Asia (KLPB), Kunming Institute of Botany, Chinese Academy of Science, Kunming, 650201 Yunnan China

Correction to: *Scientific Reports* https://doi.org/10.1038/s41598-018-36573-z, published online 21 December 2018

A Supplementary Information file containing the accession numbers for the uncultured clones was omitted from the original version of this Article. This has been corrected in the HTML and PDF version of the Article.

